# Systemic-Lupus-Erythematosus-Related Acute Pancreatitis: A Cohort from South China

**DOI:** 10.1155/2012/568564

**Published:** 2012-06-19

**Authors:** Yanlong Yang, Yujin Ye, Liuqin Liang, Tianfu Wu, Zhongping Zhan, Xiuyan Yang, Hanshi Xu

**Affiliations:** ^1^Department of Rheumatology, The 1st Affiliated Hospital, Sun Yat-Sen University, Guangzhou, Guangdong 510080, China; ^2^Department of Internal Medicine/Rheumatology, University of Texas Southwestern Medical Center, Dallas, TX 75390, USA

## Abstract

Acute pancreatitis (AP) is a rare but life-threatening complication of SLE. The current study evaluated the clinical characteristics and risk factors for the mortality of patients with SLE-related AP in a cohort of South China. *Methods*. Inpatient medical records of SLE-related AP were retrospectively reviewed. *Results*. 27 out of 4053 SLE patients were diagnosed as SLE-related AP, with an overall prevalence of 0.67%, annual incidence of 0.56‰ and mortality of 37.04%. SLE patients with AP presented with higher SLEDAI score (21.70 ± 10.32 versus 16.17 ± 7.51, *P* = 0.03), more organ systems involvement (5.70 ± 1.56 versus 3.96 ± 1.15, *P* = 0.001), and higher mortality (37.04% versus 0, *P* = 0.001), compared to patients without AP. Severe AP (SAP) patients had a significant higher mortality rate compared to mild AP (MAP) (75% versus 21.05%, *P* = 0.014). 16 SLE-related AP patients received intensive GC treatment, 75% of them exhibited favorable prognosis. *Conclusion*. SLE-related AP is rare but concomitant with high mortality in South Chinese people, especially in those SAP patients. Activity of SLE, multiple-organ systems involvement may attribute to the severity and mortality of AP. Appropriate glucocorticosteroid (GC) treatment leads to better prognosis in majority of SLE patients with AP.

## 1. Introduction

Systemic lupus erythematosus (SLE) is a chronic, autoimmune, inflammatory disease characterized by the presence of a plethora of autoantibodies, immune complex formation, and multiple organ system involvement. Gastrointestinal (GI) manifestations are common in SLE patients, but acute pancreatitis is rare [1–6]. It was reported that 19.2%–50% of SLE patients presented with gastrointestinal symptoms [7–11], whereas pancreatitis occurred in about 0.7%–8.2% of patients with SLE [7, 8, 11, 12] and the annual incidence was approximately 0.4–1.1‰ [3–5]. Our knowledge about SLE-related acute pancreatitis (AP) is mostly based on individual case reports or small case series. Despite its rarity, AP can be a life-threatening complication of SLE if not treated appropriately. Prevalence of SLE is relatively high in Chinese people, which is 0.7~1/1000 in comparison to 0.51/1000 in United States [13]. But so far very few case reports on SLE-related AP in Chinese population have been published. The current study aims to clarify the clinical characteristics, severity, mortality, and outcome of SLE-related acute pancreatitis in south China.

## 2. Materials and Methods

A retrospective review of inpatient medical records between January 2000 and January 2012 was performed at the First Affiliated Hospital of Sun Yat-Sen University in South China. 4053 patients were classified as SLE during the past 12 years who fulfilled at least four of the American College of Rheumatology (ACR) revised classification criteria for SLE (1997) [14]. A diagnosis of acute pancreatitis (AP) was established by the presence of typical clinical symptoms (including abdominal pain, nausea, and vomiting) and confirmed by more than a three-fold elevation of serum amylase or lipase or evidence of imaging findings-computer tomography [CT] scan or ultrasonography (USG) [15]. Among these SLE patients, 27 were with dual simultaneous diagnosis of AP, and another 23 age- and gender-matched SLE patients without AP were randomly selected. Review of the clinical files of these 50 SLE patients was performed and data was extracted. 

 The SLE Disease Activity Index (SLEDAI) [16] was used to evaluate SLE activity during AP, and patients were defined as active SLE if the SLEDAI score was equal to or greater than 6. The Systemic Lupus International Collaborating Clinics/ACR (SLICC/ACR) damage index [17] was used to ascertain organ damage in SLE. The Atlanta criteria [18] were used to classify the severity of acute pancreatitis. Severe acute pancreatitis (SAP) was defined as the presence of at least three of Ranson's criteria and eight or more Acute Physiology and Chronic Health Evaluation II (APACHE II) score, or with the evidence of organ failure (systolic blood pressure < 90 mmHg, PaO_2_ ≤ 60 mmHg on room air, creatinine > 2 mg/dL, gastrointestinal bleeding > 500 mL/24 h, DIC or severe hypocalcemia ≤ 7.5 mg/dL) or local complications (i.e., pancreatic necrosis, abscess, or pseudocyst). The positivity of CT scan was defined as diffuse or segmental enlargement of the pancreas, illegibility of peripancreas fat, low/high density area in contrast, and peripancreas effusion [19]. The positivity of USG was defined as pancreatic enlargement, decreased echodensity, and possible fluid collections [20]. 

Demographic information including gender, age at SLE onset, duration between the onset of SLE and AP, history of alcohol consumption, gallstone, metabolic abnormalities (hypertriglyceridemia and hypercalcemia), clinical symptoms, laboratory findings, medications (especially corticosteroid, and immunosuppressive agents (ISA)) and outcome were documented. Acute pancreatitis related to mechanical obstruction (choledocholithiasis), toxic-metabolic etiologies (alcohol intake, drugs, hypercalcemia, or hypertriglyceridemia), infection, or trauma were ruled out in every case [21].

### 2.1. Statistical Analysis

 Statistical analysis was done using the SPSS program 13.0 and Prism software version 5.0. The Mann-Whitney *U* test was used for continuous variables and the chi-square or Fisher's exact test for categorical variables. Survival rates were estimated using the Kaplan-Meier method. A *P* value <0.05 was considered statistically significant in all comparisons.

## 3. Results

### 3.1. Demographic and Clinical Characteristics of SLE-Related Acute Pancreatitis

27 out of 4053 SLE patients were diagnosed as SLE-related AP during the past 12 years, with an overall prevalence of 0.67% and annual incidence of 0.56‰. One patient developed 2 episodes of pancreatitis and the other 26 patients had only one episode at the time of hospitalization. The demographic and clinical features of each SLE-related AP patient were shown in Table 1.

### 3.2. Comparison of Demographic and Clinical Features in SLE Patients with and without SLE-Related AP

The majority of patients (92.59%, 25/27) were females and the mean age at SLE onset was 26.96 ± 13.30 years (ranged from 14 to 57 years). Time interval between the onset of SLE and AP ranged from 1 week to 20 years, and more than half of the patients (51.85%, 14/27) developed AP within the first year of the onset of SLE. All these 27 patients were classified as active SLE with average SLEDAI score of 21.70 ± 10.32 at the onset of AP. The clinical features related to acute pancreatitis in these 27 SLE patients were nonspecific. Abdominal pain (92.59%), fever (77.78%) and nausea/vomiting (74.07%), were the most frequent manifestations and other symptoms included diarrhea (44.44%), loss of appetite (44.44%) and GI tract hemorrhage (14.81%).

Other organ system involvement was found in all SLE-related AP patients with an average number of 5.70 ± 1.56 (ranged from 3 to 8 organs), including hematological system, kidney, liver, serositis, mucocutaneous involvement, respiratory system, arthritis, and central nervous system.

Clinical features and laboratory findings were compared between these two groups and the results were shown in Table 2. SLE patients with AP presented with higher SLEDAI score (21.70 ± 10.32 versus 16.17 ± 7.51, *P* = 0.03), more organ system involvement (5.70 ± 1.56 versus 3.96 ± 1.15, *P* = 0.001), higher frequence of fever (77.78% versus 39.13%, *P* = 0.006), hepatological and hematological disorders (82.61% versus 34.78%, *P* = 0.01; 100% versus 60.87%, *P* = 0.001), serositis (62.96% versus 26.09%, *P* = 0.01), elevated CRP (81.82% versus 47.62%, *P* = 0.02), positive anti-La antibody (33.33% versus 0, *P* = 0.003), and higher mortality (37.04% versus 0, *P* = 0.001) compared to SLE patients without AP.

### 3.3. Comparison of Clinical Features between SAP and MAP Patients

**Figure 1:**
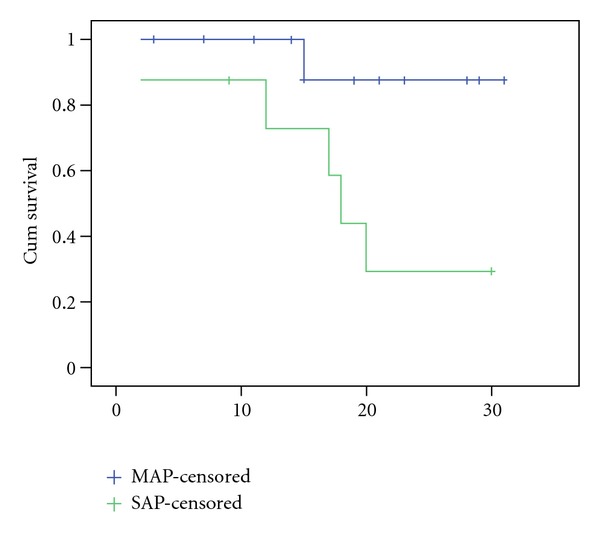
Kaplan-Meier survival curves for the time (days) from onset of SLE-related acute pancreatitis to death.

According to Atlanta criteria, 27 SLE-related AP patients were divided into SAP group (severe acute pancreatitis, *n* = 8, 29.63%) and MAP group (mild acute pancreatitis, *n* = 19, 70.37%). The comparison of the demographic and clinical data between SAP and MAP patients as shown in Table 3. The results indicated that the age of onset of AP in SAP patients as significantly younger than MAP (19.63 ± 10.88 versus 30.05 ± 13.25, *P* = 0.016). SAP patients presented with significantly higher mortality (75% versus 21.05%, *P* = 0.014) and more abnormal hematologic findings (thrombocytopenia and leucopenia, 100% versus 52.63%, *P* = 0.026; 87.5% versus 31.58%, *P* = 0.013, resp.) compared to MAP. The Kaplan-Meier survival curves showed death rate within 30 days after onset of acute pancreatitis in SAP and MAP groups (Figure 1).

### 3.4. Comparison of Clinical Features between Pediatric- and Adult-Onset SLE-Related AP

 SLE-related AP patients were divided into pediatric-onset group (under 18 years of age, *n* = 10) and adult-onset group (*n* = 17). Demographic and clinical characteristics were compared between these two groups. Pediatric-onset SLE-related AP had higher rate of severe AP (60% versus 11.76%, *P* = 0.014), higher serum amylase level (17.55 ± 16.09 versus 6.53 ± 5.42, *P* = 0.007), lower percentage of positive anti-Ro antibody (25% versus 84.62%, *P* = 0.01), and lower rate of anti-La antibody (0 versus 53.85%, *P* = 0.02) compared to adult-onset SLE-related AP. However, the difference in mortality was not statistically significant between pediatric and adult patients (50% versus 29.41%, *P* = 0.26).

### 3.5. Comparison of Clinical Features between Mortality and Nonmortality SLE Patients with AP

The risk factors for mortality were further analyzed. 27 SLE-related AP patients were divided into mortality group (*n* = 10) and nonmortality group (*n* = 17). The clinical manifestations were compared between these two groups and shown in Table 4. The mortality group had higher percentage of hypoalbuminemia (90% versus 47.06%, *P* = 0.031), hyperbilirubinemia (40% versus 5.88%, *P* = 0.047), hematuria (100% versus 41.18%, *P* = 0.002), and granular casts (70% versus 23.53%, *P* = 0.024) compared to nonmortality group. Severity of acute pancreatitis was the most powerful risk factor for mortality in SLE-related AP (OR 11.25, 95% CI (1.611, 78.57) and *P* = 0.014).

## 4. Treatment and Outcome

**Figure 2:**
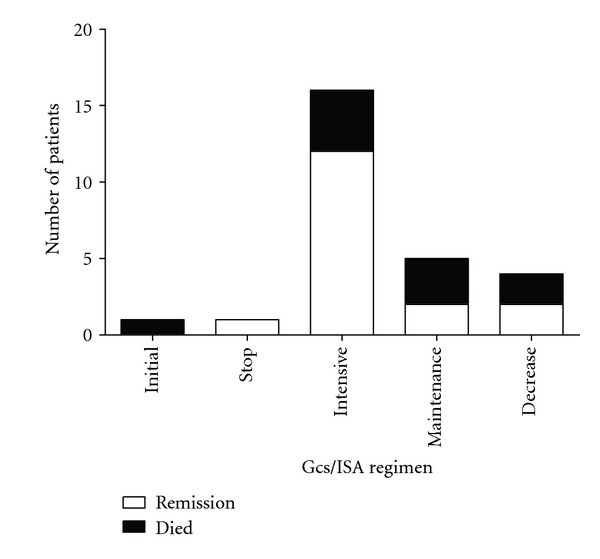
Treatment regimen and outcome of the SLE-related AP.

 Among these 27 SLE-associated AP patients, 26 were on steroid treatment before the onset of AP and the average dosage of GCs was 61.19 ± 37.63 mg/day (ranged from 10 mg/day to 120 mg/day). AP was considered as the initial presentation of SLE in one patient (patient 7 in Table 1), and standard GC treatment started after diagnosis. Additional immunosuppressive agents (ISA) were also administrated in 22 patients before the onset of AP, including 18 on hydroxychloroquine, 2 azathioprine, 8 methotrexate, 5 cyclophosphamide, and 1 FK506. After the episodes of AP, oral medicines were stopped because of fasting. Methotrexate or cyclophosphamide were continuously prescribed in 5 patients but switched to I.V. injection. 1 patient developed recurrent episode of AP when increasing the dosage of GC for the relapse of SLE, and GC treatment was stopped (patient 2) after onset of AP. 25 patients were continuously treated by GCs and/or ISA during their episode of AP. 16 patients were given aggressive treatment of GCs and/or ISA (12 patients obtained clinical and laboratory improvement (75%) and 4 died), 5 patients were treated with the maintenance dose of GCs and/or ISA (2 patients in remission (40%) and 3 died), and 4 patients were treated with decreased dose of GCs because of fever and concerning of potential infections (2 patients in remission (50%) and 2 died) (The results showed in Figure 2). Totally, 10 patients died and the overall mortality rate was 37.04% (10/27).

## 5. Discussion 

SLE-related AP is relatively rare compared to other organ injury involved in lupus. The incidence of clinical AP associated with SLE varies from 0.7 to 4% [5, 8, 12, 22], with the annual incidence of 0.4–1.1‰ [3, 4]. Most previous studies on this issue were individual case reports or small case series. So far, the Hopkins lupus cohort [12] reported the largest case series with 63 SLE-attribute pancreatitis out of 1740 SLE patients (3.5%), and a Taiwan series reported 40 out of 2976 SLE patients (1.34%). This study was the first report of the SLE-related AP in south China. In current cohort, 27 out of 4053 SLE patients were diagnosed as SLE-related AP, with the prevalence of 0.67%, and annual incidence of 0.56‰, which is comparable with the findings of previous literatures [3–5, 8, 12, 22]. 

The pathogenic mechanism of SLE-related AP is very complex and multifactors. Vascular damage (including vasculitis, intimal thickening, immune complex deposition, occlusion of arteries, and arterioles), autoantibody production, abnormal cellular immune response, and drug toxicity may be responsible for the development of pancreatitis [8]. In the current cohort, more than half patients (51.85%) developed acute pancreatitis within 1 year of the onset of SLE, and all 27 patients were active SLE with dramatically elevated SLEDAI scores and other simultaneous SLE manifestations, especially the hematologic and renal involvement. SLE patients with AP presented with higher SLEDAI scores compared to patients without AP. Previous studies [3, 4, 22, 23] also demonstrated that episodes of SLE-related pancreatitis significantly increased in the active SLE group. AP was considered as one of the clinical features of active SLE and was associated with the activity of the disease itself. These results indicated that SLE itself can be the primary etiologic factor or cofactor predisposing to AP.

 SLICC/ACR damage index score represents disease burden in SLE patients. It was significantly higher in SLE patients with pancreatitis compared to SLE patients without pancreatitis in Hopkins cohort [12]. Although SLE-related AP had more organ system involvement in current study, the damage index score was low, and there was no significant difference between SLE patients with and without AP (1.19 ± 0.92 versus 0.96 ± 1.19, *P* = 0.11). The reason of the low-damage index score might lie in the relatively younger onset age, shorter duration of disease, and less-chronic organ damage. 

Our study found that pediatric-onset AP tended to be more severe compared to adult-onset AP. SAP group had significant higher prevalence of thrombocytopenia and leucopenia than MAP group. Mortality patients has higher rate of hypoalbuminemia, hematuria, granular casts, and hyperbilirubinemia than nonmortality group, which indicated that multiple organ systems involvement, especially hematological, renal, and liver injury in SLE patients might be the major causes due to the severity and mortality of AP. In general population, the mortality rate of AP is about 3.8% ~ 10% [24–27]. Approximately 15~20% of all AP cases were SAP which accounted for a mortality rate of 16.3% ~ 30% [27–29]. SLE-related AP patients had much higher mortality. Wang et al. [23] reported that the mortality rate was 27.5% in all SLE-related AP and 78.57% in SAP. Richer et al. [30] reported that 57% of childhood-onset lupus with pancreatitis developed SAP with the mortality of 45%. In our cohort, the overall mortality rate of SLE-related AP was 37.04% compared to 0 in SLE patients without AP (*P* = 0.001), and mortality rate in SAP was 75%. The severity of AP might be the most important risk factor for the mortality of SLE-related AP patients (OR 11.25, 95% CI (1.611, 78.57), and *P* = 0.014). 

In accordance with other literatures, the manifestations of SLE-related AP in this cohort were nonspecific and similar to non-SLE acute pancreatitis. Abdominal pain (92.59%), fever (77.78%), and nausea/vomiting (74.07%) were the most common symptoms. These symptoms could also be attributed to other gastrointestinal diseases or adverse reactions of medication and may lead to misdiagnosis in general practice. It was reported that the rate of misdiagnosis of AP in SLE was up to 88.6% [31]. Delayed diagnosis and improper treatment may contribute to unfavorable prognosis, even lifethreatening [32]. Likewise, the mortality rate of the Hopkins Lupus Cohort (3%) was considerably lower than average of other reported studies due to close monitoring, early diagnosis, and treatment [12]. So, AP should be paid more attention in any SLE patient with abdominal pain when mechanical obstruction or toxic-metabolic etiologies, infection, or trauma were ruled out. 

Some immunosuppressants, such as corticosteroids, azathioprine, and cyclosporine have been implicated to cause pancreatitis in several case reports. Only 2 patients in our study took azathioprine but the medication was discontinued after the onset of AP. The current study couldn't verify the relationship between azathioprine and acute pancreatitis in SLE patients. There is still a controversy over steroid treatment in SLE-related AP. Increasingly accumulated evidence showed that steroids do not trigger acute pancreatitis or cause increased mortality on AP [22, 33, 34], but instead, they have a possible therapeutic effect on SLE-related pancreatitis [5, 35–37]. In Hopkins cohort, appropriate treatment with corticosteroids added a survival benefit in SLE-related AP. In current study, 16 SLE-related AP patients received intensive GC and/or ISA treatment, and 75% of them exhibited favorable prognosis. 

In summary, SLE-related acute pancreatitis is rare but with high-mortality rate, which is even higher in those severe acute pancreatitis with multiple organ system involvement. Activity of SLE, hematological system, renal, and liver injury in SLE patients may attribute to the mortality of AP. Early diagnosis of acute pancreatitis in SLE patients, especially those with abdominal pain, and appropriate glucocorticosteroid treatment is beneficial for a better therapeutic outcome in the majority of patients.

## Figures and Tables

**Table 1:** The demographic and clinical characteristics of each SLE patient with AP.

Case	Age at SLE onset (y)	Duration between onset of SLE and AP (m)	SLEDAI score at onset of AP	Number of involved organs concomitant with AP	GC treatment after onset of AP	Outcome
1	23	0.5	9	5	Increased dose	In remission
2	16	48	12	4	Stop	In remission
3	18	12	14	6	Increased dose	Died
4	22	72	14	7	Increased dose	In remission
5	16	36	16	7	Increased dose	In remission
6	57	24	17	4	Increased dose	In remission
7	36	0.5	17	6	Initial treatment	Died
8	48	180	18	7	maintaining	Died
9	14	36	21	4	maintaining	Died
10	14	12	23	7	Increased dose	Died
11	19	2	23	6	Increased dose	In remission
12	14	2	27	7	Increased dose	Died
13	46	1	25	4	Increased dose	In remission
14	22	0.25	18	3	Decreased dose	In remission
15	51	240	18	6	Decreased dose	In remission
16	42	4	19	7	Increased dose	In remission
17	20	84	18	5	Decreased dose	Died
18	39	24	13	5	Increased dose	In remission
19	15	2	33	6	Decreased dose	Died
20	15	36	41	7	Increased dose	In remission
21	26	3	41	8	Increased dose	Died
22	39	1	27	6	Increased dose	In remission
23	20	72	10	3	maintaining	In remission
24	36	12	38	6	maintaining	Died
25	16	2	8	3	Increased dose	In remission
26	30	48	47	8	Increased dose	In remission
27	14	72	19	6	maintaining	In remission

**Table 2:** Comparison of demographic and clinical features in SLE patients with and without AP.

	SLE with AP (*n* = 27)	SLE without AP (*n* = 23)	*P*
Female (%)	25 (92.59%)	20 (86.96%)	0.42
Age on SLE diagnosis (y)	26.96 ± 13.30	28.39 ± 9.98	0.26
GCs dose (mg)	61.19 ± 37.63	50.96 ± 28.82	0.18
SLEDAI score	21.70 ± 10.32	16.17 ± 7.51	**0.03**
SLICC/ACR damage index	1.19 ± 0.92	0.96 ± 1.19	0.11
Mortality	10 (37.04%)	0	**0.001**
Fever (%)	21 (77.78%)	9 (39.13%)	**0.006**
Neuropsychiatric (%)	7 (25.93%)	1 (4.35%)	**0.042**
Pulmonary (%)	11 (40.74%)	5 (21.74%)	0.13
Articular (%)	16 (59.26%)	16 (69.57%)	0.32
Mucocutaneous involvement (%)	18 (66.67%)	16 (69.57%)	0.54
Renal (%)	24 (88.89%)	20 (86.96%)	0.59
Hepatological (%)	19 (82.61%)	8 (34.78%)	**0.01**
Hematological (%)	27 (100.00%)	14 (60.87%)	**0.001**
Serositis (%)	17 (62.96%)	6 (26.09%)	**0.01**
Number of organs involved	5.70 ± 1.56	3.96 ± 1.15	**0.001**
Positive anti-dsDNA (%)	24 (88.89%)	19 (82.61%)	0.41
Positive anti-Sm (%)	6/21 (28.57%)	10 (43.48%)	0.24
Positive anti-Ro (%)	13/21 (61.90%)	14 (60.87%)	0.60
Positive anti-La (%)	7/21 (33.33%)	0	**0.003**
Positive ACL-IgG (%)	4/21 (19.05%)	1/22 (4.55%)	0.168
Positive ACL-IgM (%)	4/21 (19.05%)	1/22 (4.55%)	0.16
Positive anti-*β*_2_ GPI (%)	3/21 (14.29%)	2/22 (9.09%)	0.48
Low C3 (%)	26/26 (100%)	22 (95.65%)	0.47
Low C4 (%)	21/26 (80.77%)	20 (86.96%)	0.42
Elevated CRP (%)	18/22 (81.82%)	10/21 (47.62%)	**0.02**

**Table 3:** Comparison of demographic and clinical characteristics between SLE-related severe acute pancreatitis (SAP) and mild acute pancreatitis (MAP).

	SAP (*n* = 8)	MAP (*n* = 19)	*P* value
Demographic characteristics			
Female	7 (87.50%)	18 (94.74%)	0.513
Age of onset AP (y)	19.63 ± 10.88	30.05 ± 13.25	**0.016**
Interval between onset of SLE and AP (m)	23.38 ± 28.25	44.36 ± 64.73	0.822
Early AP (⩽1 year)	5 (62.50%)	9 (47.37%)	0.678
SLEDAI score at onset of AP	22.13 ± 6.24	21.53 ± 11.77	0.44
SLICC/ACR damage index	1.25 ± 0.89	1.16 ± 0.96	0.854
Number of organs involved	5.75 ± 1.28	5.68 ± 1.70	0.893
Intensive therapy of GC/ISA	5 (62.50%)	11 (57.89%)	1
Mortality	6 (75%)	4 (21.05%)	**0.014**

Clinical characteristics			
Fever	8 (100.00%)	13 (68.42%)	0.136
Mucocutaneous involvement	6 (75.00%)	12 (63.16%)	0.676
Articular involvement	3 (37.50%)	10 (52.63%)	0.678
Serositis	5 (62.50%)	10 (52.63%)	0.696
Neuropsychiatric involvement	2 (25.00%)	5 (26.32%)	1
Renal involvement	7 (87.50%)	15 (78.95%)	1.0

Laboratory findings			
Serum amylase^∗^	18.09 ± 18.15	7.46 ± 5.88	0.077
Serum lipase^∗^	8.53 ± 3.14	7.63 ± 5.45	0.616
Elevated serum transaminase	7 (87.5%)	11 (57.89%)	0.201
Thrombocytopenia	8 (100%)	10 (52.63%)	**0.026**
Leucopenia	7 (87.50%)	6 (31.58%)	**0.013**
Positive anti-dsDNA	8 (100.00%)	16 (84.21%)	0.532
Positive anti-Sm	1/7 (14.29%)	5/14 (35.71%)	0.613
Low C3	8/8 (100.00%)	18/18 (100.00%)	1
Low C4	7/8 (87.50%)	14/18 (77.78%)	1
Anti-Ro	2/7 (28.57%)	11/14 (78.57%)	0.056
Anti-La	1/7 (14.29%)	6/14 (42.86%)	0.337

^∗^Times in excess of the upper limit of normal (ULN).

**Table 4:** Comparison of demographic and clinical characteristics between mortality and non-mortality group.

	Mortality (*n* = 10)	Non-mortality (*n* = 17)	*P* value
Demographic characteristics			
Female	10 (100%)	15 (88.24%)	0.387
Age of onset AP (y)	24.10 ± 12.02	28.65 ± 14.07	0.123
SLEDAI score at onset of AP	25.00 ± 9.40	19.76 ± 10.61	0.065
Number of organs involved	6.20 ± 1.14	5.41 ± 1.73	0.167
Intensive therapy of GC/ISA	4 (40%)	12 (70.59%)	0.124

Clinical characteristics			
Fever	10 (100%)	11 (64.71%)	**0.042**
Mucocutaneous involvement	6 (60%)	12 (70.59%)	0.439
Articular involvement	6 (60%)	7 (41.18%)	0.293
Serositis	7 (70%)	8 (47.06%)	0.226
Neuropsychiatric involvement	4 (40 %)	3 (17.65%)	0.204

Laboratory findings			
Serum amylase^∗^	14.79 ± 17.34	8.15 ± 6.03	0.241
Serum lipase^∗^	6.46 ± 3.51	8.20 ± 5.58	0.368
Elevated serum transaminase	8 (80%)	10 (58.82%)	0.244
Hypoalbuminemia	9 (90%)	8 (47.06%)	**0.031**
Proteinuria	10 (100%)	12 (70.59%)	0.077
Hematuria	10 (100%)	7 (41.18%)	**0.002**
Granular casts	7 (70%)	4 (23.53%)	**0.024**
Hyperbilirubinemia	4 (40%)	1 (5.88%)	**0.047**
Positive anti-dsDNA	10 (100%)	14 (82.35%)	0.232
Positive anti-Sm	2/8 (25%)	4/13 (30.77%)	0.59
Low C3	9/9 (100%)	17/17 (100%)	1.0
Low C4	7/9 (77.78%)	14/17 (82.35%)	0.58
Anti-Ro	3/8 (37.5%)	10/13 (76.92%)	0.09
Anti-La	1/8 (12.5%)	6/13 (46.15%)	0.133

^∗^Times in excess of the upper limit of normal (ULN).

## Abbreviations

AP: Acute pancreatitis
SAP: Severe acute pancreatitis
MAP: Mild acute pancreatitis
GC: Glucocorticosteroid
ISA: Immunosuppressive agents.
